# Fibrous Dysplasia of the Jaw: Advances in Imaging and Treatment

**DOI:** 10.3390/jcm12124100

**Published:** 2023-06-17

**Authors:** Katharina Theresa Obermeier, Jens Tobias Hartung, Tim Hildebrandt, Ina Dewenter, Wenko Smolka, Eric Hesse, Florian Fegg, Sven Otto, Yoana Malenova, Anusha Abdullah

**Affiliations:** 1 Department of Oral and Maxillofacial Surgery and Facial Plastic Surgery, Ludwig Maximilians University, 80337 Munich, Germany; 2 Institute of Musculoskeletal Medicine, University Hospital, LMU Munich, Fraunhoferstraße 20, 82152 Munich, Germany; 3 Musculoskeletal University Center Munich, University Hospital, LMU Munich, Fraunhoferstraße 20, 82152 Munich, Germany

**Keywords:** fibrous dysplasia, fibro-osseous lesions, jaw, oral bone disease, GNAS1 mutation

## Abstract

A total of 7% of all benign bone lesions are diagnosed as fibrous dysplasia (FD). The symptoms of FD of the jaw range from asymptomatic to dental anomalies, pain and facial asymmetry. Due to its resemblance to other fibro-osseous bone lesions, misdiagnosis often occurs and can lead to inadequate treatment. Particularly in the jaw, this lesion does not become quiescent during puberty, making fundamental knowledge about the diagnosis and treatment of FD crucial. Mutational analysis and nonsurgical approaches offer new diagnostic and therapeutic options. In this review, we examine the advances and the difficulties of the diagnosis and the various treatment modalities of FD of the jaw in order to capture the current scientific knowledge on this bone disease.

## 1. Introduction

Fibrous dysplasia (FD) is a benign fibro-osseous lesion of the bone first described by Lichtenstein in 1938 [1]. A local form, the monostotic FD, and a systemic lesion, the polyostotic form, can be differentiated. Polyostotic FD with café-au-lait spots of the skin and hormonal imbalances is called McCune–Albright syndrome [2]. Aside from that, Mazabraud syndrome is characterized by polyostotic FD and intramuscular myxomas [3].

FD has its onset during childhood or early adolescence and usually occurs within the first or second decade of life [3]. Gnathic FD typically appears in the second or third decade, which may be due to initial misdiagnosis or a lack of symptoms [4]. Patients with polyostotic FD are typically younger at the time of diagnosis than those with monostotic FD [3]. Over 90% of the lesions are monostotic, affecting only one bone, which is only true for the mandible in the craniofacial region because FD lesions in the maxilla can cross sutures into the sphenoid, the zygoma, the base of the skull and the frontonasal bones, affecting more than one bone [2,5]. Many patients with FD of the jaw present with symptoms because FD of the jaw does not normally become quiescent after puberty, as it does in extragnathic cases of FD [2,6].

In this article, we aim to discuss the multiple diagnostic and therapeutical modalities currently described in the literature in order to show recent advances that can help improve clinical practice.

## 2. Epidemiology and Clinical Appearance

FD accounts for 2.5% of all bone lesions and approximately 7% of all benign bone tumors [7]. As many patients are asymptomatic and are discovered incidentally after oral radiography imaging, determining the incidence of FD is difficult [8]. However, the maxilla is the most usually affected bone in the craniofacial area, followed by the mandible and the frontal bone [9,10]. The majority of the lesions are unilateral and occur in the posterior region of the maxilla and mandible [7,11]. Monostotic FD is more common than polyostotic FD [12].

Craniofacial FD is typically characterized by a slow-growing mass that can be painful and cause facial asymmetry [13,14]. Clinically, the lesions can be classified as quiescent (stable), nonaggressive (slow growing) or aggressive (rapid growth ± pain, pathologic fractures, malignant transformations, etc.) [15]. As a result, the presence of symptoms and the degree of impairment can be affected. The implications for life quality, in particular, have yet to be assessed.

The first symptom is usually the painless growth of the affected bone, manifesting as facial asymmetry [7,16]. Malocclusion is another symptom of mandibular FD [12]. The progression of FD often tapers off as patients reach puberty; however, cases with continuous active disease have been reported [12,17]. In adulthood, FD can be reactivated, for example, during pregnancy [2,18].

Complications, such as pathological fractures, facial paralysis and malignant transformations, are very rare [15,19]. Rapid growth of FD of the maxilla and mandible, on the other hand, may produce airway obstruction due to the posterior displacement of the tongue [12]. Involvement of the skull base may also affect the optic canal and cause subsequent blindness as a rare complication [2].

Oligodontia, enamel hypoplasia, enamel hypomineralization, attrition and tooth rotation and displacement are examples of dental anomalies in FD [2,13,20]. The infraorbital nerve and the inferior alveolar nerve may be involved in the lesion, as decompression of the inferior alveolar nerve resulted in pain relief in a patient with mandibular FD [21]. Furthermore, alternations in the nerve canals were found in some patients, implying that patients with FD of the jaw need evaluations of the function of the inferior alveolar nerve and the infraorbital nerve [21].

## 3. Pathophysiology and Mutational Analysis

FD is caused by a somatic postzygotic missense mutation in codon 201 of GNAS1, which codes for G_s_α [22]. Arginine is replaced by histidine at codon 201 of GNAS1 exon 8 (R201H) [23]. Rare mutations with a cysteine substitution (R201C) and mutations in codon 224 of exon 9 are described in a few cases [22,23]. G_s_α is activated, which increases the intracellular levels of cyclic adenosine monophosphate (cAMP) in osteoprogenitor cells and inhibits their differentiation into mature osteoblasts [24,25]. The elevation of cAMP alters the transcription and expression of some target genes, such as c-fos, a proto-oncogene [3]. A high abundance of c-fos was detected in FD-affected bones compared to normal, uninvolved bones obtained from patients with FD [3].

Whether the mutation leads to monostotic and local or polyostotic and generalized manifestation depends on the onset of the mutation during embryogenesis [4]. Earlier mutations lead to a polyostotic disease, whereas later mutations result in focal monostotic lesions [4].

The detection of the mutation is not always reliable because somatic mosaicism causes the coexistence of mutant and wild-type GNAS 1 within the lesion, making detection using PCR or sequencing approaches ineffective. The prevalence of the mutation may be higher in active or polyostotic lesions, while it may be lower in stable and monostotic FD lesions [26].

Xue et al. (2022) demonstrated that the mutational analysis of GNAS can help to differentiate between FD and other lesions, such as chronic diffuse sclerosing osteomyelitis or ossifying fibroma [27]. They also show that out of 29 patients diagnosed with FD, only 24 (83%) had detectable mutations [28]. Overall, the positive rate for GNAS1 mutation in the craniofacial bones is around 78% [27]. As the percentage of the mutated cells decreases with age and standard PCR and sequencing require a mutant threshold of 20% to identify the mutation, this level of sensitivity may not be reached [27]. This implies that considering the mosaic features of FD, diagnosis cannot be ruled out when no mutation is found [27]. The dynamic mosaic consists of mutant and nonmutant cells, with mutant cells gradually normalizing and sterilizing in older lesions due to a greater level of apoptosis in the mutated cells relative to nonmutant cells [23].

Many patients have not been genetically characterized to determine whether the absence of the G_s_α mutation raises the risk of aggressive behavior [12].

## 4. Diagnostic Findings

Due to similarities with other fibro-osseous lesions, FD diagnosis is difficult in clinical practice [29,30]. FD of the jaws differs from other bones radiologically and histologically, possibly because of its desmal origin [2]. FD in the craniofacial area may vary from other areas because head and neck lesions are poorly demarcated, while axial lesions may be well circumscribed [31].

### 4.1. Radiographic Features

**Figure 1:**
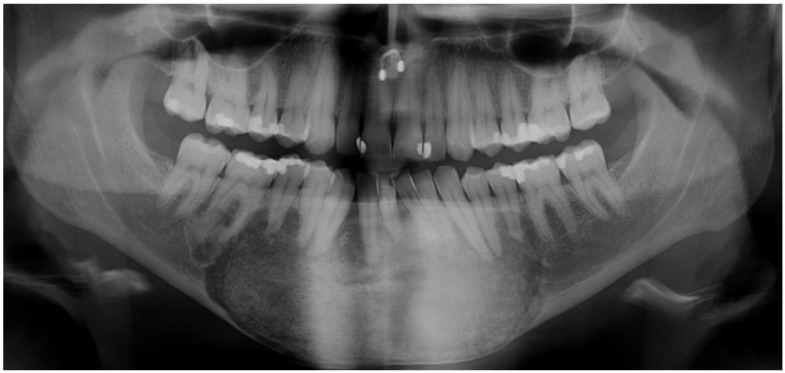
Panoramic radiography of FD in the anterior mandible. The characteristic features of FD of the jaw, such as ground-glass appearance and thin borders, are represented.

Panoramic radiography can be utilized as a primary diagnostic tool, although CT imaging is required to determine the extent of the disease [13,29,32,33,34]. One feature of craniofacial FD is a ground-glass appearance with a thin cortex and without distinct borders (Figure 1) [35]. FD can be detected via CT in three varieties: a ground-glass pattern (56%), a homogenously dense pattern (23%) and a cystic pattern (21%) [13].

It may vary with a homogenous appearance or a mixed radiopaque/radiolucent lesion as the disease progresses [12,13]. Initially the radiological alternation starts with radiolucency and turns into a more mixed stage with a radiolucent and radiopaque structure before finally becoming radiopaque [18,36,37]. Soluk-Tekessin et al. describe different radiological shape variations (septa and multilocularity) for the mandible. Lesions are generally solitary and unilateral and can cross the midline of the mandible. Resorption of teeth is rarely seen, but teeth displacement is possible. FD lesions have poorly defined and blended borders [33]. The radiological findings are characterized by peripheral “blending” and poorly defined borders between dysplastic and normal bone [37]. Thin cortices and bone expansion can be seen using CT [38]. 

Lesions in the mature stage may appear to have septa, and as a result of this multilocular appearance, the bone appears thin with irregularly positioned trabeculae [13]. 

The shift in CT appearance corresponds to increased FD activity via rapid growth or malignant transformation [12]. Therefore, Lee et al. (2012) recommend the monitoring of FD and intermitted craniofacial CT during the pubertal phase.

In FD lesions, MRI shows reduced and nonspecific signal intensity [13,14]. Bone scintigraphy can be used to determine whether or not a lesion is metabolically active [39].

As all benign fibro-osseous tumors go through calcification maturation stages, their radiological appearances may be similar [37]. Cordeiro et al. used lacunarity analysis to characterize FD, concluding that FD CT scans have lower lacunarity than normal bone, indicating that their texture images are more homogenous [32]. 

FD lesions can expand not only on the external surface but also on the internal surface, leading to expansion into orbital and nasal cavities, fissures, fossae, neural canals and maxillary sinus obliteration [40]. Furthermore, FD of the mandible might displace the inferior alveolar canal superiorly or inferiorly [3,41]. Dental MRI may be an adequate method for detecting nerve continuity and displacement without exposure to radiation. However, further studies are needed to evaluate whether dental MRI could be used as a diagnostic tool in the future.

### 4.2. Histomorphology

Another diagnostic tool to confirm the diagnosis of FD is bone biopsy. It is debatable whether a biopsy is required in asymptomatic and quiescent lesions [12]. Furthermore, histology provides no predictive or prognostic information [12]. However, biopsies can be used to rule out other pathologies in active cases [42].

The microscopic diagnosis of FD is also difficult due to overlapping features with other fibro-osseous lesions [38,43]. FD shows a cellular collagenous stroma without mitotic figures and pleomorphism. Evenly distributed blood vessels and elongated trabeculae of woven or lamellar bone with irregular curves (often described as the Chinese letters pattern) are observed [38].

Differentiation between FD and ossifying fibroma can be carried out by the clear separation of the lesion from the normal bone in ossifying fibroma, whereas in FD, a close association of the lesioned bone and the normal bone with blending in some areas can be observed [38].

Shmuly et al. demonstrated that FD lesions have significantly more vascularization than other fibro-osseous bone diseases [42].

Immunohistochemistry can be used to detect elevated levels of osteocalcin, a bone formation marker, as well as OPG, a protein that inhibits bone resorption and antagonizes RANKL in FD [44,45]. Differences in the expression of osteocalcin, in particular, can assist in distinguishing between ossifying fibroma and FD [45].

### 4.3. Differential Diagnosis and Malignant Transformation

The current classification of head and neck tumors by the World Health Organization (5th edition) combines odontogenic and maxillofacial bone tumors, including bone and chondral tumors, fibro-osseous tumors and dysplasia. FD is subdivided into monostotic FD and polyostotic FD, including McCune–Albright syndrome and Jaffé–Lichtenstein syndrome. Another entity in this group is ossifying fibroma [46].

The differential diagnosis between fibro-osseous lesions is difficult due to overlapping clinical, radiological and histopathological characteristics [47]. A potential biomarker in the diagnosis of FD could be the GNAS1 gene mutation, which is a variable parameter due to genetic mosaicism with mutated and wild-type GNAS1 within the lesion [47]. Pannone et al. investigated the role of the Wnt/β- catenin pathway, which is upregulated due to G_s_α activating mutations. Elevated signaling can lead to downstream gene transcription and may inhibit osteoblast maturation [38]. Studies have also shown how the detection of positive nuclear ß-catenin excludes FD during differential diagnosis because most of the FD were negative for nuclear staining [47,48].

Differences between FD and ossifying fibroma were found based on DNA copy number analysis with microdissection sequencing, which detected distinct copy number alternations in patients with FD and ossifying fibroma [49]. The differentiation of FD and ossifying fibroma is necessary because of varying treatment modalities, as ossifying fibroma is a true neoplasm and needs complete enucleation because of its recurrence risk [30,45].

**Figure 2:**
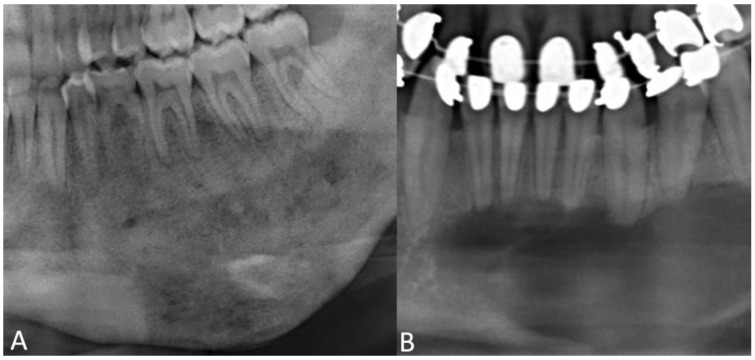
An excerpt of a panoramic radiography of a patient with chronic diffuse sclerosing osteomyelitis (**A**) and of a patient with central giant-cell granuloma of the mandible (**B**). (**A**) shows broad zones of sclerosing and subperiosteal bone formation. (**B**) shows well-defined and undulating borders.

Another differential diagnosis of FD can be chronic diffuse sclerosing osteomyelitis of the mandible, which appears as fibroinflammatory tissue histologically and shows broad zones of sclerosis radiologically (Figure 2) [50,51]. Cortical lysis and subperiosteal bone formation are more common in patients with chronic diffuse sclerosing osteomyelitis, whereas FD radiologically presents with bone expansion and mandibular canal displacement [50]. Another difference is the recurrence of pain every few weeks or months and the soft-tissue swelling in chronic diffuse sclerosing osteomyelitis, which is not described for FD [50]. Osteoblastoma and central giant-cell granuloma should also be considered for differential diagnosis as they can mimic fibro-osseous lesions [51].

In infants, FD should be differentiated from cherubism which usually presents as bilateral jaw swelling and the characteristic upward turn of the eye [52]. Cherubism defines another entity of bone disease due to its SH3BP2 mutation and mainly affects osteoclastogenesis, while FD affects the osteoblasts [53].

All types of FD can transform into sarcomas and most commonly into osteosarcomas, but malignant transformations into fibrosarcoma, chondrosarcomas and angiosarcomas have also been reported [2,54,55,56]. Significant rapid growth and change in the radiographic appearance suggest malignancy. Malignant transformation is relatively rare, occurring in 0.4% to 4% of cases [10,33,54]. Potential risk factors for the malignant transformation of FD are radiotherapy, polyostotic FD, McCune–Albright syndrome and an excess of growth hormone [54]. GNAS1 and TP53 mutations have been detected in the malignant transformation of FD into osteosarcoma [55].

## 5. Treatment Options

### 5.1. Surgical Approaches

In 1990, Chen and Noordhoff developed an approach for the therapy of FD in different craniofacial zones [9,57]. They advised conservative treatment and not to excise the teeth-bearing areas of the maxilla and the mandible, which is Zone 4 according to their classification; though in cases of overgrowth of the maxilla, it may be necessary to perform total excision and reconstruction with bone grafts [57]. In the fronto-orbital and the maxillozygomatic (Zones 1 and 2) complex, they adopted extensive surgery because of the high occurrence of hypertelorism, dystopia and other complications [57].

Treatment modalities of FD of the jaw range from watchful waiting to minimal or extensive surgery depending on the progression of the disease [9]. Early studies recommend extensive surgery and immediate reconstruction of FD in the maxilla and the mandible because no recurrence is expected [58]. Nevertheless, the surgery should be performed when skeletal maturity has been achieved, and the lesion has reached a static phase [9,30,59,60].

At present, the latest studies lean towards watchful waiting for stable cases because wide resections require reconstruction, which is associated with higher postoperative morbidity compared to surgical shaving [15,61]. One disadvantage of minimal surgical treatment through bone shaving is the higher recurrence rate [58]. In terms of surgical procedures, minimal surgery with shaving or debulking is nowadays carried out more often than radical resections [9].

With computer-assisted navigation, minimal surgical therapy can result in better aesthetic and functional results. Digital templates can be useful for preserving the inferior alveolar neurovascular bundle in the surgical treatment of FD [62]. Complete resection of the lesion requires a reconstruction, and patients with FD of the jaw may need orthognathic surgery to correct a malocclusion [12]. Furthermore, regular follow-up is obligatory to identify recurrence [15,63].

The quality of life after surgical treatment for FD has been evaluated, and both conservative and radical surgical therapy reduce the scores for activity and speech, but the total resection also led to declined chewing function [64].

Annual evaluations of quiescent FD lesions with sensory nerve testing in the involved region and facial CT are recommended [12].

### 5.2. Nonsurgical Approaches

Medical treatment of FD with bisphosphonates to arrest bone resorption is used to reduce symptoms and lesion growth [9,65]. Studies have presented mixed results on the efficiency of bisphosphonates in FD pain relief [66,67]. These studies mostly include FD lesions in long bone pain, not the craniofacial sites [12]. However, there is no evidence that bisphosphonates influence lesion progression or the activity of FD [68,69]. It has been discussed whether bisphosphonates are not efficient because their action needs incorporation into the mineralized matrix, which is decreased in FD [68].

Pamidronate can be given intravenously to reduce osteoclastic activity and decrease the intensity of bone pain and bone resorption [1,70]. Studies have shown that oral alendronate may not be effective in the treatment of bone pain [71]. Otto et al. (2015) observed promising results of single-shot ibandronate infusions in the pain relief of patients with diffuse sclerosing osteomyelitis [72]. Further studies are needed to assess the efficiency of oral or intravenously administered bisphosphonates and the required dose for sufficient pain relief in patients with FD of the jaw. 

The incidence of MRONJ (medication-related osteonecrosis of the jaw) in FD patients treated with antiresorptives is low, which implies a low risk of side effects by the use of bisphosphonates in adult and pediatric patients with FD [73,74,75,76,77].

RANKL (receptor activator of the nuclear factor κ-Β ligand) is excessively abundant in patients with FD and has led to the use of denosumab, which is a monoclonal antibody that binds RANKL and inhibits the connection to its receptor RANK [78]. This leads to suppression of the stimulation of osteoclasts and, therefore, of bone resorption. Studies on mouse models showed that denosumab induces the formation of mineralized bone within lesions and prevents the progression of FD, but it is also described that lesions reoccurred after therapy with potentially life-threatening hypercalcemia [78,79,80,81]. Meier et al. suggest restricting the use of denosumab to cases in which bisphosphonates are not tolerated or not effective. Potential rebound phenomena after withdrawal from denosumab should also be considered and need further investigation [24].

Radiotherapy leads to a higher risk of developing sarcoma and should, therefore, not be used in the treatment of FD [1,82].

Alkaline phosphatase (ALP) is a marker for bone formation by osteoblasts [83]. The serum concentration of alkaline phosphates in individuals with FD can occasionally be higher than in other craniofacial fibro-osseous lesions [84]. The role of ALP as a prognostic marker is controversial, but it positively correlates with the extent of the disease, particularly in polyostotic FD compared to monostotic FD [38]. Onyebuchi et al. advised the monitoring of serum ALP in the treatment of patients with FD of the craniofacial region to detect disease recurrence [84,85].

## 6. Conclusions

The diagnosis of FD is challenging due to radiological and histological overlaps with other fibro-osseous diseases of the jaw. Analysis of the GNAS1 mutation can help to differentiate between FD and other jaw bone disorders. However, somatic mosaicism may hamper the detection of the mutation in some cases. An increased apoptosis of mutant cells compared to nonmutant cells, as well as their decreased prevalence in older and stable FD, suggest that FD cannot be ruled out when the mutation is not detected. We propose that age-dependent bone morphology changes in FD of the jaw should also be taken into account during the diagnosis process. 

The identification of the GNAS1 mutation opens up new ways of treatment. As, so far, only symptomatic treatment options for FD are available, targeted therapy after mutational analysis of the patients could be considered in the future. 

## Data Availability

The data used to support the content of this study are included within the article.
